# Long-term outcomes after upfront second-generation tyrosine kinase inhibitors for chronic myeloid leukemia: managing intolerance and resistance

**DOI:** 10.1038/s41375-024-02187-w

**Published:** 2024-02-29

**Authors:** Simone Claudiani, Farhan Chughtai, Afzal Khan, Chloe Hayden, Fiona Fernando, Jamshid Khorashad, Victoria Orovboni, Glenda Scandura, Andrew Innes, Jane F. Apperley, Dragana Milojkovic

**Affiliations:** 1 Centre for Haematology, Department of Immunology and Inflammation, Faculty of Medicine, Imperial College London, London, UK; 2 Department of Haematology, Hammersmith Hospital, Imperial College Healthcare NHS Trust, London, UK; 3 Imperial Molecular Pathology, Hammersmith Hospital, Imperial College Healthcare NHS Trust, London, UK

**Keywords:** Chronic myeloid leukaemia, Targeted therapies

## Abstract

Second-generation tyrosine kinase inhibitors (2GTKI) are more effective in inducing rapid molecular responses than imatinib when used first-line in patients with chronic myeloid leukemia in chronic phase (CML-CP). However, failure of first line-2GTKI (1L-2GTKI) still occurs and there is no consensus regarding subsequent management. We retrospectively analyzed the outcome of 106 CML-CP patients treated with 1L-2GTKI and with a median follow-up of 91 months. 45 patients (42.4%) switched to an alternative TKI, 28 for intolerance (26.4%) and 17 (16%) for resistance. Most patients who remained on 1L-2GTKI achieved deep molecular responses (DMR) and 15 (14.1%) are in treatment-free remission (TFR). Intolerant patients also obtained DMR, although most required multiple TKI changes and were slower to respond, particularly if treated with 2L-imatinib. Inferior outcomes were observed in resistant patients, who failed alternative 2L-2GTKI and required 3/4GTKI and/or allogeneic hematopoietic stem cell transplant (alloSCT). 7yr-OS was significantly lower for these individuals (66.1%) than for intolerant patients and those who remained on 1L-2GTKI (100% and 97.9%, respectively; *p* = 0.001). It is apparent that failure of 1L-2GTKI is a challenging problem in modern CML therapy. Intolerance can be effectively managed by switching to an alternative 2GTKI, but resistance requires early consideration of 3/4GTKI.

## Introduction

Second-generation TKI (2GTKI) used in first line (1 L) allow more rapid achievement of molecular responses in patients in CML-CP, compared to 1L-imatinib-treated patients [1–3]. The advantages of 1L-2GTKI include increased efficacy in CML patients with high-risk prognostic scores [4] and in those in whom TFR is the primary therapeutic goal. Resistance to upfront 2GTKI is less common than on imatinib [5] and there is a paucity of information regarding management strategies. So far, there have been no head-to-head comparisons of 2GTKI versus third-generation TKI (3GTKI) for patients failing an upfront 2GTKI. Current CML treatment guidelines do not provide definitive recommendations on the optimal therapeutic choice in the setting of 1L-2GTKI failure. In this context, the new Specifically Targeting ABL Myristoyl Pocket (STAMP)-inhibitor, asciminib, has strengthened the therapeutic armamentarium for intolerant/resistant CML patients and could soon emerge as a beneficial treatment option in the second line (2 L) setting.

Our study contributes to the current information regarding the management of CML patients who fail an upfront 2G-TKI, offering extended follow-up and a detailed analysis of subsequent treatment pathways.

## Methods

We analyzed the long-term outcomes of patients treated with a 1L-2GTKI between January 2007 and July 2022. The study was approved by our internal review board and written informed consent for data collection and analysis was obtained from all patients. Nilotinib and dasatinib were approved for 1 L use in United Kingdom in 2012. All patients who received these TKI prior to 2012 did so within the ENESTnd [1], ENEST1st [6] and SPIRIT2 [7] studies. All bosutinib-treated patients were included in the BFORE study [3].

Inclusion criteria were CML in chronic phase (CP), age ≥18 years at diagnosis and treatment with upfront 2GTKI. The choice of first and subsequent lines of therapy at our center was in accordance with the recommendations of the British Society of Haematology Guidelines and the ELN [8, 9].

We analyzed achievement of cytogenetic and molecular responses irrespective of the line of TKI therapy, incidence and timing of 1L-2GTKI switch due to intolerance or resistance, progression to accelerated (AP) or blast phase (BP) at any time after commencement of 1L-2GTKI therapy, event-free survival (EFS), progression-free survival (PFS), overall survival (OS) and achievement of treatment-free remission (TFR). Standard (ELN 2020) definitions of responses and disease progression were used [9]. A *BCR::ABL1*/*ABL1* RT-qPCR ratio ≤1% (MR2) was considered to be equivalent to complete cytogenetic response (CCyR) [10]. Deep molecular response was defined as MR4 (*BCR::ABL1*/*ABL1* RT-qPCR ratio ≤ 0.01%) or deeper.

For the analysis of cumulative probabilities of response, the date of first achievement of response was used, regardless of which TKI line the patient was on at that time. Cumulative rates of response were also separately described for each TKI line.

Survival analysis was by the method of Kaplan-Meier. For EFS, we considered events to be failure of 1L-2GTKI due to any cause, disease progression to AP/BP or death; patients were censored at last follow-up on 1L-2GTKI. For PFS, events recorded were progression to AP/BP or death; censoring was performed at last follow-up on TKI. For OS, events were death for any cause (CML-related and CML-unrelated). For all survival analyses the starting date was that of the CML diagnosis.

Treatment-free remission was defined as being alive off TKI and in at least major molecular response. For the probability of TFR we included only patients with at least 6 months follow-up in TFR and all the patients who lost MR3 at any time. All analyses were performed with SPSS software (version 25; IBM, USA) and GraphPad Prism software (version 8).

## Results

### Overall outcomes after starting 1L-2GTKI

**Fig. 1:**
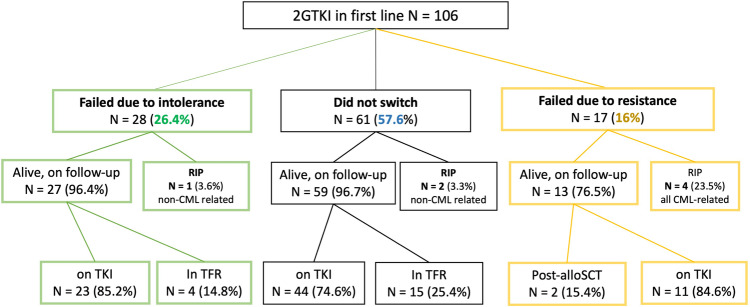
Overall treatment outcome in 106 CML patients after starting a 1L-2GTKI. The figure shows the long-term outcome in the whole cohort of CML patients in 1st chronic phase (*N* = 106) treated with an upfront second-generation TKI (2GTKI) at a single center. RIP deceased patients, alloSCT allogeneic hematopoietic stem cell transplant, TFR treatment-free remission.

The baseline characteristics of 106 patients are provided in Table 1. After a median follow-up of 91 months (8–183), 61 patients (57.6%) remained on 1L-2GTKI, 28 switched for intolerance (26.4%) and 17 patients for resistance (16%)(Fig. 1).

**Table 1:** Patients’ baseline characteristics.

	ALL (*n* = 106)	No switch (*n* = 61)	Intolerant (*n* = 28)	Resistant (*n* = 17)
**Gender, Female (%)**	58 (54.7)	34 (55.7)	16 (57.1)	8 (47.1)
**Median age (range)**	38.8 (18–75)	36.5 (19–75)	43.4 (22–64)	40.9 (18–70)
**Sokal**				
- *low, n (%)*	37 (34.9)	24 (39.3)	11 (39.3)	2 (11.8)
- *intermediate*	24 (22.6)	11 (18)	7 (25)	6 (35.3)
- *high*	27 (25.5)	14 (23)	7 (25)	6 (35.3)
- *unknown*	18 (17)	12 (19.7)	3 (10.7)	3 (17.6)
**ELTS**				
- *low, n (%)*	52 (49.1)	31 (50.8)	15 (53.6)	6 (35.3)
- *intermediate*	23 (21.7)	13 (21.3)	6 (21.4)	4 (23.5)
- *high*	13 (12.3)	5 (8.2)	4 (14.3)	4 (23.5)
- *unknown*	18 (17)	12 (19.7)	3 (10.7)	3 (17.6)
***BCR::ABL1*** **transcript type**				
* - E14a2*	42 (39.6)	24 (39.3)	9 (32.1)	9 (52.9)
* - E13a2*	38 (35.8)	20 (32.8)	13 (46.4)	5 (29.4)
* - E14a2-e13a2*	16 (15.1)	8 (13.1)	5 (17.9)	3 (17.6)
* - unknown*	10 (9.4)	9 (14.8)	1 (3.6)	0
**First-line 2GTKI**				
* - Dasatinib*	53 (50)	29 (47.5)	16 (57.1)	8 (47.1)
* - Nilotinib*	46 (43.4)	27 (44.3)	10 (35.7)	9 (52.9)
* - Bosutinib*	7 (6.6)	5 (8.2)	2 (7.1)	0
**Median follow-up, months (range)**	90.9 (8–183)	97.3 (8–183)	95.5 (10.9–171.5)	72.5 (9.8–151.7)
**Median duration of 1st** **line therapy, months (range)**	36.6 (0.1–182.7)	73 (8–182.7)	13.8 (0.1–107.3)	11 (3.4–68.5)

The total number of patients who achieved each response level on 1L-2GTKI was: 82 of 106 patients (77.3%) for CCyR, at a median of 3.4 months (1–14.5); 76 (71.7%) for MR3, at a median of 5.7 months (2.8–36.7); 65 for MR4 (61.3%), at a median of 13.8 months (3–63); 55 for MR4.5 (51.9%), at a median of 19.9 months (4.6–107); 38 for MR5 (35.8%), at a median of 59.8 months (8.6–153). Supplementary Table 1 provides more details regarding the achievement of each response level in patients who failed their 1L-2GTKI. Among 61 who continued 1L-2GTKI, 44 remain on 1L-2GTKI (72.1%), 2 patients (3.3%) died while in chronic phase (one each of T-cell prolymphocytic leukemia and a cardiac event) and 15 (24.6%) are in TFR at last review. Evaluating the 61 patients who continued on 1L-2GTKI, 61 (100%) achieved CCyR, 58 (95%) MR3, 52 (85.2%) MR4, 46 (75.4%) MR4.5 and 37 (60.6%) MR5 at a median of 3.6 (1–11), 5.5 (3–36.7), 14 (3–59), 21.3 (5–107) and 61.9 (8.6–153) months, respectively.

**Fig. 2:**
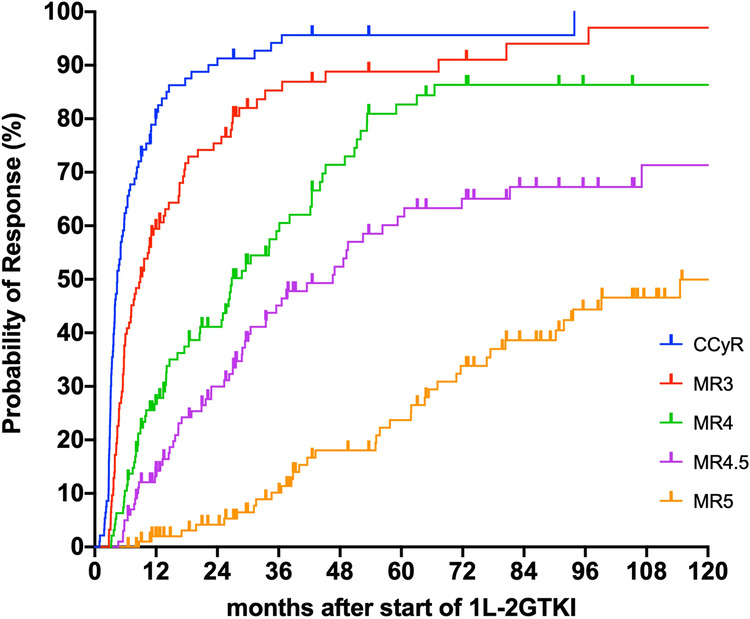
Cumulative probabilities of response after starting a 1L-2GTKI. The figure shows the cumulative probabilities of achievement of each response level after commencing an upfront second-generation TKI (1L-2GTKI) in CML patients in first chronic phase. The *x* axis indicates the time from the 1L-2GTKI start to the date of response, regardless to which TKI line this was achieved on. CCyR Complete cytogenetic response.

One-year (1-yr) and 3-yr cumulative probabilities of responses, regardless of which TKI line they were achieved on, were: 81.3% and 94.6%, 59.6% and 85.3%, 26.7% and 59%, 14.2% and 45.1% and 2% and 9.1% for CCyR (MR2), MR3, MR4, MR4.5 and MR5, respectively (Fig. 2). The median time to achievement of each level of response was 4.1 (1–94), 7 (2.8–96.6), 17.5 (3–66.5), 22.8 (4.6–107) and 56.8 months (8.6–153), for CCyR, MR3, MR4, MR4.5 and MR5, respectively.

**Fig. 3:**
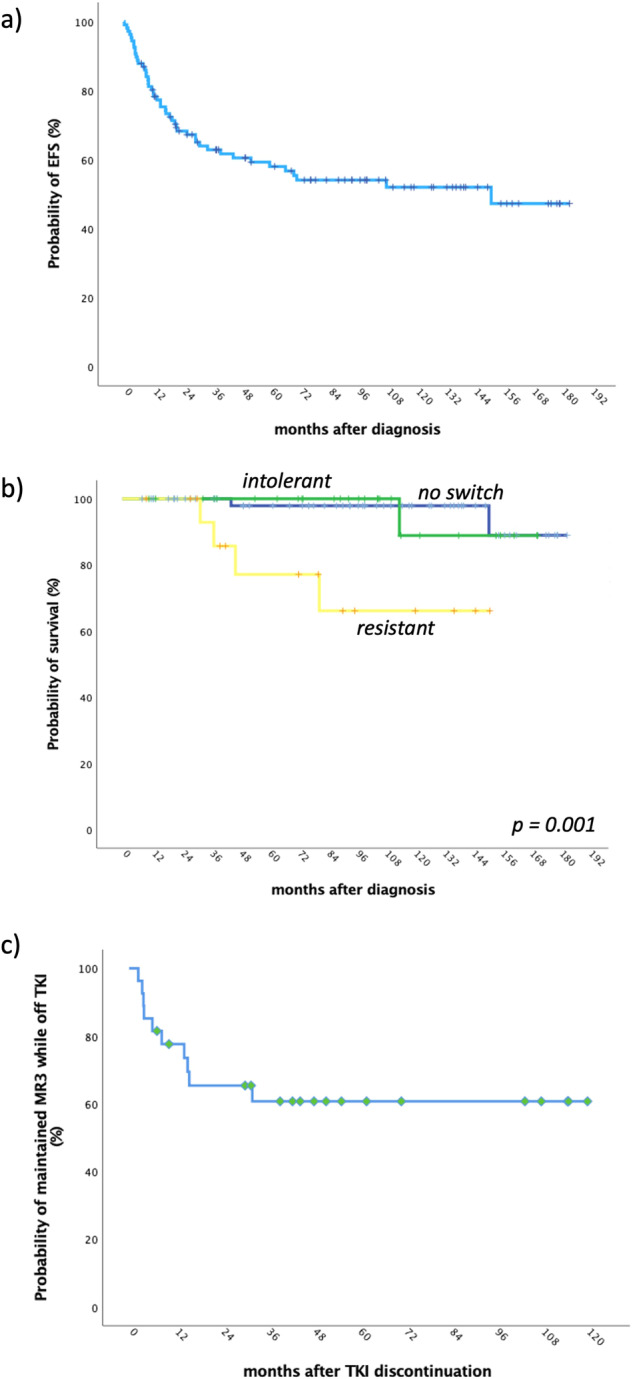
Long-term outcomes (EFS, OS and TFR) in 106 patients treated with 1L-2GTKI. Panel **a** shows the probability of event-free survival for the entire patient cohort. The *x* axis indicates the time from diagnosis until the occurrence of any event (failure of 1L-2GTKI due to any cause, disease progression to accelerated (AP)/blast phase (BP), death) or censoring, done at last follow-up on 1L-2GTKI. Panel **b** shows the probability of overall survival for each patient group: those who remained on 1L-2GTKI (no switch, *n* = 61, blue line), those who failed their 1L-2GTKI due to intolerance (intolerant, *n* = 28, green line) and the resistant patients (resistant, *n* = 17, yellow line). The *x* axis indicates the time from diagnosis until the occurrence of death from any cause or censoring, done at last follow-up on TKI. The *p*-value reported on the right bottom of the Kaplan-Meier plot refers to the log-rank test. Panel **c** shows the probability treatment-free remission for 28 patients at their first TFR attempt (after discontinuation of 1L-2GTKI and ≥2L-TKI in 22 and 6 patients, respectively). The *x* axis indicates the time from TKI discontinuation until the occurrence of MR3 loss or censoring, done at last follow-up in MR3 while off TKI.

The 5-yr and 7-yr EFS were 58% (95% CI: 48.1–67.3%) and 54.1% (43.9–63.9%), respectively (Fig. 3a), while 5-yr and 7-yr PFS were 95.3% (95% CI: 88.5–98.2%) and 93.9% (95% CI: 86.3–97.4%), respectively.

The 7-yr OS of the overall population was 93.8% (95% CI: 86.2–97.3%). Looking at each patient group, the 7-yr OS was 97.9% (95% CI: 88.9–99.6%), 100% and 66.1% (95% CI: 37.4–86.4%) for those who did not switch, the intolerant and the resistant patients respectively (*p* = 0.001, log-rank test; Fig. 3b).

### Intolerant patients

The median follow-up of the intolerant patients is 95.5 months (10.9–171.5 months) with a median time to switch to 2L-TKI of 13.8 months (0.1–107.3) and a median follow-up after failure of 1L-2GTKI of 81.7 months.

Sixteen of 53 (30.2%), 10 of 46 (21.7%) and two of 7 patients (28.6%) discontinued 1L-dasatinib, 1L-nilotinib and 1L-bosutinib, respectively. Toxicities leading to discontinuation of each 1L-2GTKI are reported in Table 2.

**Table 2:** Toxicities leading to discontinuation of 1L-2GTKI.

	Dasatinib (*n* = 16 patients)	Nilotinib (*n* = 10 patients)	Bosutinib (*n* = 2 patients)
***Hematological***			
	(*n* = 3)	(*n* = 1)	(*n* = 1)
***Cardiovascular***			
	Congestive heart failure (*n* = 1)	Palpitations and chest pain (*n* = 1)	
	Pericarditis (*n* = 1)	Palpitations, chest pain and hypertension (*n* = 1)	
		Peripheral arterial occlusive disease (PAOD) (*n* = 1)	
***Pulmonary***			
	Pulmonary arterial hypertension and pleural effusion (*n* = 1)		
	Shortness of breath (*n* = 2)		
***Gastrointestinal and Hepatic***			
	Gastrointestinal (*n* = 1)		
	Hepatic (*n* = 1)	Hepatic (*n* = 1)	Hepatic (*n* = 1)
***Metabolic***			
		Diabetes (*n* = 1)	
***Dermatological***			
	Skin rash (*n* = 1)	Skin rash (*n* = 1)	
		Alopecia (*n* = 1)	
***General***			
	Fatigue (*n* = 2)	Fatigue (*n* = 1)	
	Migraine (*n* = 1)		
***Other***			
	Retinal hemorrhage (*n* = 1)	Thyroiditis (*n* = 1)	
	Follicular hyperplasia (*n* = 1)		

Seventeen patients received 2L-imatinib (60.7%) and 11 (39.3%) a 2L-2GTKI. Responses at the time of starting 2 L therapy were: unknown (*n* = 2), <MR3 (*n* = 11), MR3 or deeper (*n* = 4) in the 2L-imatinib group; <MR3 (*n* = 3) and MR3 or deeper (*n* = 8) in the 2L-2GTKI group. Switching to 2L-imatinib was unsuccessful in the short-term in the majority due to a combination of intolerance and lack/loss of molecular responses (Supplementary Table 2a): only 2/17 (11.8%) patients who switched to 2L-imatinib did not require a switch to 3 L therapy compared to 5/11 (45.4%) in the 2L-2GTKI group. Furthermore only 1/11 (9.1%) patients in the latter group required ≥4L therapy, compared to 5/17 (29.4%) who received 2L-imatinib. All four patients who switched to 2L-imatinib for hematological toxicitydeveloped subsequent resistance; ten of eleven patients with non-hematological intolerance to 1L-2GTKI failed 2L-imatinib for intolerance.

Ten of 17 (58.8%) of the patients who switched to 2L-Imatinib switched to an alternative TKI at least once more because of intolerance; of whom two (20%) due to the persistence of the adverse event (AE) from the 1L-2GTKI, two (20%) due to the combination of persistent and new AEs and six (60%) due to the appearance of new toxicities. Of the latter, three patients required a switch to ≥4 L due to further intolerance (Supplementary Table 4a).

In the 2L-2GTKI group, six of 11 (54.5%) patients switched to an alternative TKI at least once more due to intolerance, of which five had resolved the AEs experienced on their 1L-2GTKI and one had persistent AE. All these patients required 3L-TKI due to new toxicities; of them, one had to discontinue their 3L-TKI due to intolerance, achieving TFR, and one required up to 6L-TKI (Supplementary Table 4b).

Most patients (25, 89.3%) achieved MR3, with 22 going on to deeper response (78.6%). Rates of achievement of each response level are provided in Supplementary Table 1. For those not in MR4 at the start of 2L-TKI (*n* = 21), achievement of MR4 was faster on 2L-2GTKI than on 2L-imatinib: 15 months (1.4–90) versus 34.6 months (12.6–101.2), respectively (*p* = 0.025).

Three of 6 patients who discontinued treatment achieved TFR at the first attempt: all had received a 2L-2GTKI. One of the three patients who failed TFR at the first attempt, achieved it on a second attempt after stopping 5L-imatinib. Supplementary Fig. 1 and Supplementary Table 2a and 2b illustrate the treatment line switches and outcomes for the 1L-2GTKI-intolerant patients.

Of the 28 intolerant patients, 23 (82.1%) continue on TKI, four are in TFR (14.3%) and one (3.6%) experienced sudden death on 3L-nilotinib, 9.5 years from diagnosis.

At last follow-up, the response levels were <MR3, MR3, and DMR in two (11.8%), five (29.4%), 10 (58.8%) and none, two (18.2%) and nine (81.8%) patients of the 2L-Imatinib and 2L-2GTKI groups, respectively. Progression to AP/BP was not observed in any of the patients and no patient received alloSCT.

### Resistant patients

The median follow-up of the 17 resistant patients was 72.5 months (9.8–151.7), with a median time to switch to 2L-TKI of 11 months (3.4–68.5) and a median follow-up after failure of 1L-2GTKI of 61.5 months.

Primary resistance occurred in 14 (82.3%) (lack of MR1 at 6 months or MR2 at 12 months; only 1 case of lack of MR3 after 21 months): secondary resistance was noted in three (17.7%) at 19.7 and 68 months from 1L-nilotinib (N) and 60 months after 1L-dasatinib. The level of response at switch to 2L-therapy was CHR in three, MR1 in 11 and CCyR in three patients.

After 1L-2GTKI failure, 2L-therapy was an alternative 2G-TKI for 11 patients, ponatinib for four, imatinib for one and alloSCT for another patient with a T315I mutation.

Five of 17 patients (29.4%) who switched their 1L-2GTKI because of resistance switched TKI at least once more due to intolerance (please refer to Supplementary Table 4c; Supplementary Table 5 offers the AE profile in patients receiving 3/4GTKI).

After starting 2L-therapy, 1-yr and 2-yr cumulative probabilities of achieving MR3 were 33% and 54.1%, respectively. TKI at achievement of MR3 after 1L-2GTKI failure was ponatinib (*n* = 3, of whom two on 2L-ponatinib and one on 3L-ponatinib), nilotinib (*n* = 3, of whom twopatients subsequently switched TKI because intolerance or secondary resistance after achievement of at least MR3), dasatinib (*n* = 1) and asciminib (*n* = 1).

Time to MR3 was 13.8 months and 6.2 months for two of four patients who started on 2L-ponatinib, while the median time to MR3 was 19.6 months in those who received a 2L-2GTKI or 2L-imatinib. In the latter group, only two patients are still on a 2G-TKI at last follow-up; of the other 10 patients, five underwent intensive treatment (chemotherapy, *n* = 1 or alloSCT, *n* = 4) for persistent resistance in first chronic phase or progression, one achieved MR3 on 3L-ponatinib, one CCyR on 3L-asciminib, two stable MR3 and one MR4.5 on 5L- and 4L-asciminib, respectively, after multiple TKI changes due to intolerance or resistance.

All patients were screened for a *BCR::ABL1* kinase domain (KD) mutation at the time of 1L-2GTKI failure. Mutations were found in 4 patients (23.5%), i.e. one each of T315I, V299L, F359I, G250E.

Of the 17 who switched for resistance, 13 patients survive (76.5%), of whom 11 (84.6%) are on TKI and two (15.4%) are post-alloSCT. Four patients (23.5%) died of CML-related causes: one myeloid blast crisis refractory to chemotherapy at 2.75 years from starting 1L-nilotinib, one from lymphoid blast-crisis at 7 years after 1L-nilotinib and two patients due to transplant-related mortality. Of note, all progressions and deaths occurred in the 2L-2GTKI group.

Supplementary Fig. 2 and Supplementary Table 3a and 3b illustrate the treatment line switches and the outcomes in the resistant group.

### Treatment-free remission

A total of 28 patients attempted TFR, of whom 22 interrupted their 1L-TKI and 6 discontinued their 2L- (*n* = 2), 3L- (*n* = 3) and 6L-TKI (*n* = 1) and belonged to the intolerant group. No patients attempted TFR after resistance to 1L-2GTKI.

The median durations of TKI therapy, DMR and the median follow-up of maintained MR3 after TKI discontinuation were 6.8 years (2.4–11.4), 4.6 years (1.4–10.3), 4.3 years (0.6–10), respectively.

Ten patients lost MR3 at a median time of 7.3 months (2.4–32.3) from TKI discontinuation. The 3-yr probability of TFR was 60.7% (95% CI: 41.3–77.2%; Fig. 3c) in the whole group, 64.7% (95% CI: 41.9–81.5%) in those who discontinued their 1L-2GTKI and 50% (95% CI: 18.8–81.8%) in the intolerant patients (*p* = 0.46).

When separately analyzing those patients who discontinued their 1L-2GTKI (*n* = 22), the median duration of TKI therapy, of DMR and the median follow-up of maintained MR3 after TKI discontinuation were 7.2 years (2.4–11.4), 5.2 years (1.7–10.3), 4.2 years (0.6–10), respectively. Median time to MR3 loss for 7 relapsing patients was 8.5 months (3.4–32.3).

## Discussion

First-line second-generation TKI induce molecular responses rapidly in the majority of CML patients in chronic phase and are employed by many physicians if there are concerns about progression or conversely a desire to achieve the criteria for discontinuation of treatment. Our cohort is representative of these treatment decisions with a median age of 38.8 years and 35% of low Sokal risk. However, as for patients treated upfront with imatinib, we show that the need to give 2L-treatment is not uncommon, with >40% of patients needing to switch TKI, more commonly for intolerance than resistance. The rate of 1L-2GTKI failure in our patients was similar to that reported in ENESTnd (~40%) [1] and DASISION (~35%) [2]. In a EUTOS population study [11], 77 of 372 patients (20.7%) treated with upfront 2GTKI changed to 2L-therapy due to intolerance (11%), resistance (4.8%), combination of the two (0.8%) or unspecified reasons (4%). Of note, imatinib was the most common 2L-treatment for intolerant patients (58.5%), a similar finding to that reported in the global observational study, SIMPLICITY, from the analysis of the first year of 1L-TKI therapy [12]. In all the above studies, however, data on the long-term outcome of these patient subgroups are limited, especially regarding the chance of success and the number of subsequent TKI switches.

Furthermore, the use of 3G- and 4G-TKI as 2 L after failure of upfront 2GTKI has not been formally investigated and much of the available information relates to their use in third or subsequent lines, most commonly after 1L-imatinib. In a retrospective chart review of CML patients failing 1L-2GTKI at four Canadian centers [13], 76 patients (32.8%) switched to 2L-therapy and were followed up for a median of 53 months after TKI failure. The first TKI change was mostly due to intolerance (*n* = 60, 25.9%) and, less frequently than in our study, for resistance (*n* = 16, 6.9%). In the latter group, all received 2L-2GTKI (*n* = 14) or 2L-Imatinib (*n* = 2): eight patients achieved major molecular response, of which seven (44%) on a 2L-TKI. In this subgroup, one patient received alloSCT while in MMR and another died of acute coronary syndrome after having been switched to 2L-nilotinib for a V299L mutation. Of the other eight resistant subjects, one experienced progression in to blast phase CML and died after alloSCT, one died of another cancer, three required alloSCT and three never achieved an adequate response. Similar to our findings, survival was inferior in the resistant patients compared to the intolerant ones (5-year OS 80% versus 95.2%, respectively).

Although resistance to 1L-2GTKI is uncommon, being ~13% and ~11% in ENESTnd and DASISION, respectively, we identified resistance in 16%, which may reflect our selection of patients for 1L-2GTKI. We demonstrated that a switch to an alternative 2L-2GTKI is ineffective in ~75% of cases and optimal results are achieved only in those who received a 3/4GTKI. Only 2 of 11 patients who switched to 2L-2GTKI are currently in DMR on 2L-therapy, one of whom had a KD mutation that was sensitive to an alternative 2GTKI. It has to be acknowledged, however, that our study has the important limitations of a relatively small sample size and the retrospective nature, so it may not be possible to generalize these observations. However, our findings are in line with a metanalysis of clinical studies exploring the efficacy of alternate 2GTKI or ponatinib after failure of one or more prior 2GTKI [14]. Of 11 studies analyzed, the authors found that in 3L-TKI treatment the sequential use of 2GTKI was of limited value: the probability of CCyR was 22–26% compared to 60% in the ponatinib-treated patients. Similarly, a retrospective multicenter study comparing the efficacy of ponatinib versus 2GTKI in 3 L therapy in 354 CML patients, including also PACE [15] and OPTIC [16] participants, found that 3GTKI allowed a higher rate of deeper responses, longer PFS and OS and was the only independent factor associated with better survival in a propensity score matching analysis [17]. The efficacy of ≥3L-ponatinib after failure of 2GTKI was also shown by another recent sub-analysis of the PACE and OPTIC studies [18]. Interestingly, in our cohort asciminib was the most frequent therapy at last follow-up in patients who switched 1L-2GTKI due to resistance and none of these subjects required alloSCT. Our data would support the use of both ponatinib and asciminib in second line. Asciminib has been previously shown to maintain or improve molecular responses in a significant proportion of CML patients who have failed a number of TKIs, including ponatinib [19]. Data from the ASCEMBL study [20] clearly showed a benefit for asciminib 40 mg bd over bosutinib 500 mg od in third or subsequent line treatment in terms of major molecular response rates and long-term tolerability.

Conversely, in intolerant patients, switching to an alternative 2GTKI seems to be a reasonable option in most patients, whereas 2L-imatinib resulted in a higher number of treatment changes, longer times to achieve deep molecular responses and a lower likelihood of TFR. In the above-mentioned Canadian study [13], 31 of 60 intolerant patients (51.6%) received 2L-imatinib, compared to the 60.7% in our study, but most of these patients did not require further switches. The response level at first TKI switch was not provided for this subgroup, so we do not know whether these patients had deeper responses at the start of imatinib in comparison to our patients. We also observed that the reason for 2L-imatinib failure was more likely to be persistent intolerance rather than resistance.

Despite the significant proportion of 1L-2GTKI failure, we showed that overall survival of CML patients remains high for those without signs of resistance to upfront treatment. In contrast, all CML-related deaths occurred in the group of resistant patients.

Finally, our study is the first to report ‘real-life’ data on 1L-2GTKI treated patients inclusive of TFR achievement outside TFR clinical trials [21, 22]. In our cohort, TFR was achieved by around a quarter of patients who started and continued on a 1L-2GTKI and this goal was also possible in a significant, although smaller, proportion (14.8%) of patients failing 1L-2GTKI upfront due to intolerance. The higher TFR probability in our study, compared to DASFREE [22] and ENESTfreedom [21] studies could be related to the longer exposure to 1L-2GTKI in our cohort.

As mentioned above, the main disadvantages of our study are its retrospective nature and the relatively small patient cohort analyzed. However, to the best of our knowledge, our work offers the longest follow-up after failure of 1L-2GTKI and the advantage of being monocentric, which ensures homogeneity of clinical data and patients’ management [23]. For these reasons, the present study contributes to the increasing pool of evidence and helps to delineate the best practice in the management of this challenging scenario in CML treatment pathways.

## Conclusion

Failure of 1L-2GTKI represents a clinical challenge that mandates an expert consensus on the best treatment strategy, especially in the resistant patients and in the era of low-dose ponatinib regimens and the availability of the new STAMP inhibitor asciminib. Patients who are intolerant to 1L-2GTKI might be sufficiently well-managed with an alternative 2GTKI. Although confirmation of our findings must come from other and larger studies, our work sheds light on the long-term outcome of 1L-2GTKI treated patients and offers management strategies.

### Supplementary information

Supplementary Material

## Data Availability

All data generated or analyzed during this study are included in this published article and its supplementary information files.
