# Supplementary material for: Long-term outcomes after upfront second-generation tyrosine kinase inhibitors for chronic myeloid leukemia: managing intolerance and resistance
Source: Leukemia. 2024 Feb 29;38(4):796–802. doi: 10.1038/s41375-024-02187-w (PMC10997507; doi:10.1038/s41375-024-02187-w)
Supplement: Supplementary file 1 — Supplementary Material [file 41375_2024_2187_MOESM1_ESM.docx]

**Supplementary Material**

**Long-term outcomes after upfront second-generation tyrosine kinase inhibitors for chronic myeloid leukemia: managing intolerance and resistance**

Authors: Simone Claudiani, Farhan Chughtai, Afzal Khan, Chloe Hayden, Fiona Fernando, Jamshid Khorashad, Victoria Orovboni, Glenda Scandura, Andrew Innes, Jane F. Apperley, Dragana Milojkovic

**List of contents**

**Supplementary Table 1.**

**Rate of responses in patients who failed 1L-2GTKI, by TKI line on which the response was achieved for the first time….…………………………….............................................................page 3**

**Supplementary Figure 1.**

**Treatment pathways and outcomes after 1L-2GTKI failure for intolerance in 28 CML patients…………………………………………………………………………………………...page 4**

**Supplementary Table 2a.**

**Treatment pathways in 17 CML patients who switched to 2L-imatinib due to intolerance to 1L-2GTKI……………………………………………………………………………………………page 5**

**Supplementary Table 2b.**

**Treatment pathways in 11 CML patients who switched to alternative 2L-2GTKI due to intolerance to 1L-2GTKI………………………………………………………………………..page 6**

**Supplementary Figure 2.**

**Treatment pathways and outcomes after 1L-2GTKI failure for resistance in 17 CML patients…………………………………………………………………………………………...page 7**

**Supplementary Table 3a.**

**Treatment pathways in 13 CML patients without KD mutations at failure of 1L-2GTKI due to resistance…………………………………………………………………………………………page 8**

**Supplementary Table 3b.**

**Treatment pathways in 4 CML patients with KD mutations at failure of 1L-2GTKI due to resistance…………………………………………………………………………………………page 9**

**Supplementary Table 4a.**

**Subsequent TKI changes due to intolerance after 2L-imatinib (n = 10 patients).……………………………………………………………………………………….page 10**

**Supplementary Table 4b.**

**Subsequent TKI changes due to intolerance after 2L-2GTKI (n = 6 patients)…..……………………………………………………………………………………page 11**

**Supplementary Table 4c.**

**Subsequent TKI changes due to intolerance after resistance to 1L-2GTKI (n = 5 patients) .………………………………………………………………………………………page 12**

**Supplementary Table 5.**

**Adverse event profile in patients receiving 3/4GTKI…….……………………………page 13**

**Supplementary Table 1.**

**Rate of responses in patients who failed 1L-2GTKI, by TKI line on which the response was achieved for the first time.**

| **Response** | **Line** | **Switched for INTOLERANCE**  **(n=28)** | | | **Switched for RESISTANCE**  **(n=17)** | | |
| --- | --- | --- | --- | --- | --- | --- | --- |
|  |  | n (%) | median t., mo | Total, n (%) | n (%) | median t., mo | Total, n (%) |
| CCyR | 1L | 16 (57.1) | 3.34 | 26 (92.9) | 5 (29.4) | 4.4 | 14 (82.3) |
|  | 2L | 2 (7.1) | 8.5 |  | 4 (23.5) | 11.1 |  |
|  | 3L | 6 (21.4) | 12.2 |  | 2 (11.8) | 25 |  |
|  | 4L | 1 (3.6) | - |  | 3 (17.6) | 31.3 |  |
|  | 5L | 1 (3.6) | - |  | 0 | - |  |
| MR3 | 1L | 15 (53.6) | 7.33 | 25 (89.3) | 3 (17.6) | 5.7 | 10 (58.8) |
|  | 2L | 0 | - |  | 3 (17.6) | 26.8 |  |
|  | 3L | 7 (25) | 17.7 |  | 2 (11.8) | 40.9 |  |
|  | 4L | 2 (7.1) | 22.1 |  | 1 (5.9) | - |  |
|  | 5L | 1 (3.6) | - |  | 1 (5.9) | - |  |
| MR4 | 1L | 11 (39.3) | 11.7 | 22 (78.6) | 2 (11.8) | 9.75 | 5 (29.4) |
|  | 2L | 3 (10.7) | 51.3 |  | 1 (5.9) | - |  |
|  | 3L | 6 (21.4) | 38.1 |  | 0 | - |  |
|  | 4L | 2 (7.1) | 32.72 |  | 1 (5.9) | - |  |
|  | 5L | 0 | - |  | 1 (5.9) | - |  |
| MR4.5 | 1L | 7 (25) | 11.3 | 13 (46.4) | 2 (11.8) | 19.39 | 3 (17.6) |
|  | 2L | 0 | - |  | 0 | - |  |
|  | 3L | 5 (17.9) | 48.6 |  | 0 | - |  |
|  | 4L | 0 | - |  | 1 (5.9) | - |  |
|  | 6L | 1 (3.6) | - |  | 0 | - |  |
| MR5 | 1L | 1 (3.6) | - | 2 (7.1) | 0 | - | 1 (5.9) |
|  | 6L | 1 (3.6) | - |  | (4L) 1 (5.9) | - |  |

Legend to Supplementary Table 1: median t.=median time to response from start of 1L-2GTKI; mo=months; 1L=1^st^ line; 2L=2^nd^ line; 3L=3^rd^ line; 4L=4^th^ line; 5L=5^th^ line; 6L=6^th^ line.

**Supplementary Figure 1. Treatment pathways and outcomes after 1L-2GTKI failure for intolerance in 28 CML patients.**


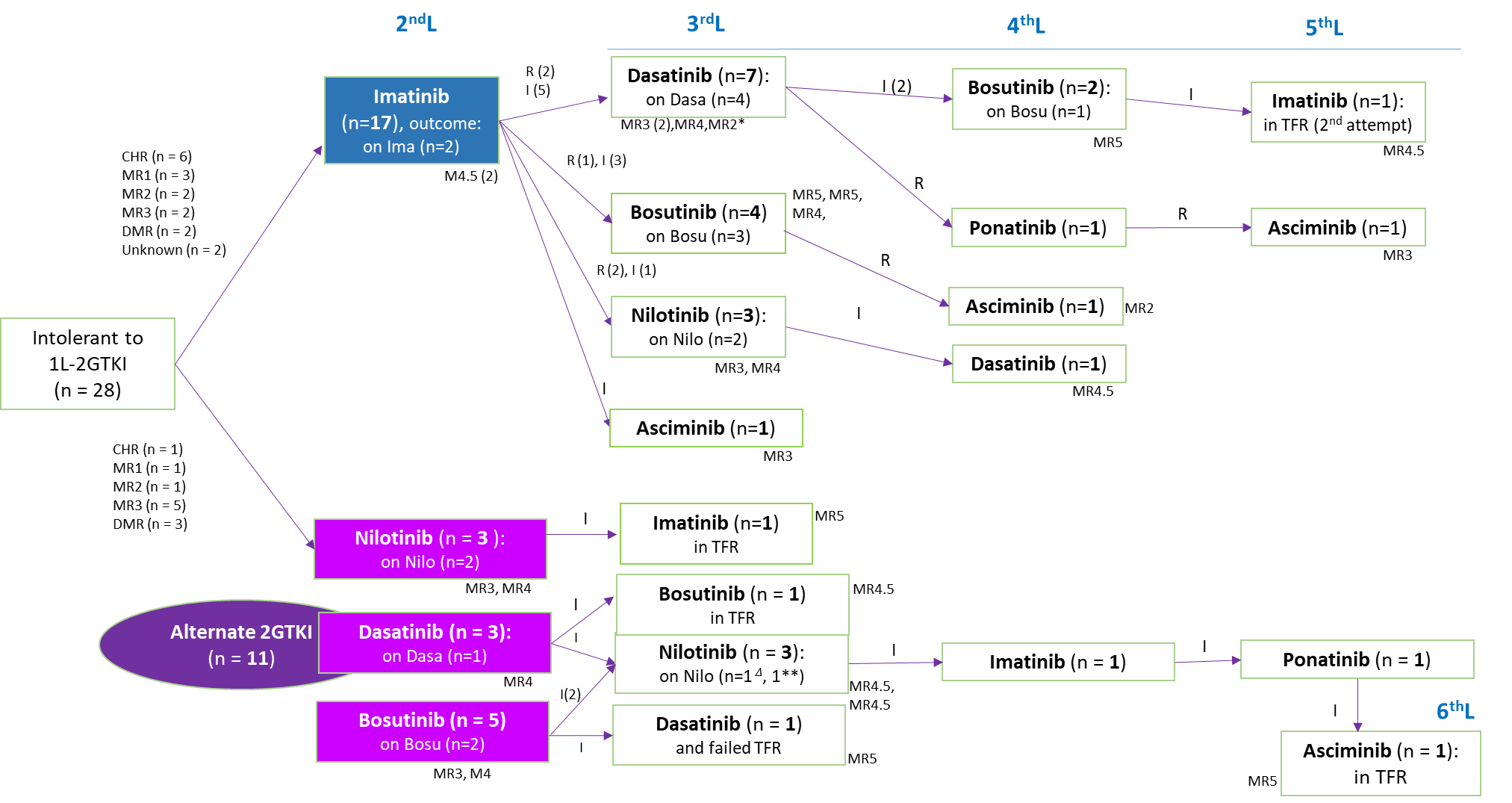


**Legend to Supplementary Figure 1**: 1L-2GTKI=1^st^ line second-generation tyrosine kinase inhibitor; CHR=complete hematological response; DMR=deep molecular response; TFR=treatment-free remission; Nilo=nilotinib; Dasa=dasatinib; Bosu=bosutinib; L = line; **deceased patient due to sudden death while in MR4.5 on nilotinib; *1 patient was in MR2 and not compliant to low-dose dasatinib; **^𝛥^** 1 patient received: nilotinib-dasatinib-nilotinib. Close to each box is indicated the response at last follow-up for each subject. Numbers in brackets on each arrow indicate the number of patients experiencing resistance (R) or intolerance (I) at each switch. Above and below the first box on the left are listed the response levels at the start of 2L-imatinib and 2L-2GTKI, respectively.

| ID | 1L | Type of intolerance (HEM or NON-H) | Response at switch | 2L | Response at switch; reason f.s. | 3L | Response at switch; reason f.s. | 4L | Response at switch; reason f.s. | 5L | Response at switch; reason f.s. | 6L | Response at switch; reason f.s. | Response at last fu |
| --- | --- | --- | --- | --- | --- | --- | --- | --- | --- | --- | --- | --- | --- | --- |
| 10 | D | HEM | CHR | I | No MR2 aft 12/12; Res | N |  | | | | | | | MR3 |
| 11 | D | NON-H (congestive heart failure) | CCyR | I | Failed TFR, then restarted on imatinib | | | | | | | | | MR4.5 |
| 14 | D | NON-H (fatigue) | MR3 | I | MR3; Into | D^*^ |  | | | | | | | CCyR (MR2) |
| 17 | D | NON-H (fatigue) | MR1 | I |  | | | | | | | | | MR4.5 |
| 27 | D | NON-H (pericarditis) | MR5 | I | MR3; Into | B |  | | | | | | | MR5 |
| 38 | D | NON-H (rash, itching) | MR1 | I | No MR1 aft 6/12; Into | N | Only 2/52 tx; Into | D |  | | | | | MR4.5 |
| 41 | N | NON-H  (alopecia) | MR4.5 | I | MR2 on LD ima; Into, failed 1^st^ TFR attempt | D | MR3; Into | B | MR2; Into | I |  | | | MR4.5  (in TFR, 2^nd^ attempt) |
| 43 | N | NON-H  (rash) | CHR | I | CHR; Into | D | MR4; Into | B |  | | | | | MR5 |
| 46 | N | NON-H  (palp., chest pain and HTN) | MR3 | I | MR2 aft stopping; Into | B |  | | | | | | | MR4.5 |
| 47 | N | NON-H  (liver) | MR1 | I | No MR3 after 10/12; Res | D |  | | | | | | | MR3 |
| 48 | N | HEM | Unknown | I | No MR3 aft 2.5y; Res | D | No MR3 aft 1.5y; Res | P | No MR3 aft 1.5y; Res | A |  | | | MR3 |
| 57 | B | HEM | CHR | I | No MR2 aft 8/12; Res | N |  | | | | | | | MR4 |
| 67 | B | NON-H  (liver) | CHR | I | No MR2 aft 8/12; Into | D |  | | | | | | | MR4 |
| 79 | D | NON-H  (SOB) | CCyR | I | MR2; Into | B |  | | | | | | | MR4 |
| 82 | D | HEM | CHR | I | No MR1 at 1.5y; Res | B | No MR3 aft 2y; Res | A^∆^ |  | | | | | CCyR (MR2) |
| 92 | N | NON-H  (palp. and chest pain) | Unknown | I | MR2; Into | D |  | | | | | | | MR3 |
| 103 | D | NON-H (liver) | CHR | I | CHR; Into | A |  | | | | | | | MR3 |

**Supplementary Table 2a.**

**Treatment pathways in 17 CML patients who switched to 2L-imatinib due to intolerance to 1L-2GTKI.**

**Legend to Supplementary Table 2a**: 1L=first line; 2L=second line; 3L=third line; 4L=fourth line; 5L=fifth line; 6L=sixth line; tx=therapy; *=poor compliance to low-dose dasatinib; ∆= only 3 months of therapy so far; Reason f.s.=clinical reason for TKI switch; CHR=complete hematological response; CCyR=complete cytogenetic response; D=dasatinib, N=nilotinib, B=bosutinib, I=imatinib, P=ponatinib, A=asciminib; Into=intolerance; Res=resistance; TFR=treatment-free remission; HEM=hematological adverse event; NON-H=non-hematological adverse event; palp.=palpitations; HTN=hypertension; liver=hepatic adverse event; SOB=shortness of breath; 12/12=12 months; 10/12=10 months; 8/12=8 months; 6/12=6 months; 2/52= 2 weeks; LD=low dose; Ima=imatinib; Nilo=nilotinib; y=years; aft=after; fu=follow-up.

**Supplementary Table 2b.**

**Treatment pathways in 11 CML patients who switched to alternative 2L-2GTKI due to intolerance to 1L-2GTKI.**

| ID | 1L | Type of intolerance (HEM or NON-H) | Response at switch | 2L | Response at switch; reason f.s. | 3L | Response at switch; reason f.s. | 4L | Response at switch; reason f.s. | 5L | Response at switch; reason f.s. | 6L | Response at switch; reason f.s. | Response at last fu |
| --- | --- | --- | --- | --- | --- | --- | --- | --- | --- | --- | --- | --- | --- | --- |
| 1 | D | NON-H  (GI) | MR3 | B | MR1; Into | N |  | | | | | | | MR4.5 |
| 9 | N | NON-H (PAOD) | MR3 | D | MR4; Into | B |  | | | | | | | MR4.5  (TFR) |
| 16 | D | NON-H (retinal h.) | MR4 | N | MR4.5; Into | I |  | | | | | | | MR5  (TFR) |
| 58 | N | NON-H (NIDDM) | MR4.5 | D | MR4.5; Into | N |  | | | | | | | MR4.5 |
| 64 | N | NON-H (thyroiditis) | CCyR | B | MR2; Into | D | Failed TFR, then restarted on dasatinib | | | | | | | MR5 |
| 75 | D | NON-H (pleural effusion, PAH, SOB) | MR3 | B |  | | | | | | | | | MR4 |
| 76 | D | NON-H  (SOB) | MR1 | B | MR1; Into | N | MR1;  Into | I | MR2; Into | P | MR2;  Into | A |  | MR5  (TFR) |
| 77 | D | HEM | MR3 | B |  | | | | | | | | | MR3 |
| 86 | N | NON-H  (fatigue) | MR3 | D |  | | | | | | | | | MR4 |
| 90 | D | NON-H  (follicular hyperplasia) | MR4 | N |  | | | | | | | | | MR4 |
| 101 | D | NON-H  (migraine) | CHR | N |  | | | | | | | | | MR3 |

**Legend to Supplementary Table 2b**: 1L=first line; 2L=second line; 3L=third line; 4L=fourth line; 5L=fifth line; 6L=sixth line; D=dasatinib, N=nilotinib, B=bosutinib, I=imatinib, P=ponatinib, A=asciminib; Into=intolerance; Res=resistance. HEM= hematological adverse event; NON-H=non-hematological adverse event; GI=gastro-intestinal; PAOD=peripheral arterial occlusive disease; NIDDM=non-insulin-dependent diabetes mellitus; retinal h.=retinal hemorrhage; PAH=pulmonary arterial hypertension; SOB=shortness of breath; TFR=treatment-free remission; fu=follow-up.

**Supplementary Figure 2. Treatment pathways and outcomes after 1L-2GTKI failure for resistance in 17 CML patients.**

**Legend to Supplementary Figure 2**: 2GTKI=second-generation tyrosine kinase inhibitor; RIP=deceased patients; 2L=2^nd^ line; 3L=3^rd^ line; 4L=4^th^ line; 5L=5^th^ line; alloSCT=allogeneic hematopoietic stem cell transplant; DMR=deep molecular response; KD mut=*BCR::ABL1* kinase domain mutation.

**Supplementary Table 3a. Treatment pathways in 13 CML patients without KD mutations at failure of 1L-2GTKI due to resistance.**

| ID | 1L | Resistance | Resp at switch (IS) | 2L | Best resp to 2L  (IS) | Outcome | 3L  (n=10) | Outcome | 4L  (n=6) | outcome | 5L  (n=2) | Outcome |
| --- | --- | --- | --- | --- | --- | --- | --- | --- | --- | --- | --- | --- |
| 20 | Dasatinib | Primary | 9.1% | **Ponatinib** | 0.023% | MR3 |  |  |  |  |  |  |
| 93 | Dasatinib | Primary | 5% | **Ponatinib** | 0.038% | MR3 |  |  |  |  |  |  |
| 96 | Dasatinib | Primary | 16.6% | **Ponatinib** | 16.59% | No MR1 after 6 months; intolerance | Dasatinib | No MR1 after 6 months | Asciminib | MR2 |  |  |
| 106 | Dasatinib | Primary | 20.3% | **Ponatinib** | 17.49% | Intolerance after  1 month | Asciminib | MR2 |  |  |  |  |
| 22 | Nilotinib | Secondary (loss of MR4.5, MR4, MR3 and MR2) | 4.9% | **Dasatinib** | 4.39% | No MR1 after 6 months | Ponatinib | Progression to BC | CT 🡪 alloSCT | RIP (relapsed/refractory lymphoid BC) |  |  |
| 36 | Dasatinib | Primary | 3.8% | **Nilotinib** | 0.71% | MR4.5 |  |  |  |  |  |  |
| 49 | Nilotinib | Primary | 4.9% | **Bosutinib** | 4.76% | No MR1 after 6 months | alloSCT | RIP (TRM) |  |  |  |  |
| 65 | Nilotinib | Primary | 9.65% | **Dasatinib** | 11.58% | No MR1 after 6 months | Ponatinib | MR1 loss | alloSCT | RIP (TRM) |  |  |
| 66 | Nilotinib | Primary | 0.32% | **Dasatinib** | 0.31% | No MR3 after 18 months | Ponatinib | MR3 |  |  |  |  |
| 74 | Nilotinib | Primary | 7% | **Dasatinib** | 2.29% | No MR2 after 12 months | Asciminib | MR2 |  |  |  |  |
| 85 | Nilotinib | Primary | 8.7% | **Dasatinib** | 8.79% | No MR2 after 12 months | Ponatinib | MR1 loss; intolerance | alloSCT | Asciminib post-alloSCT, MR4.5 |  |  |
| 87 | Dasatinib | Primary | 2.8% | **Bosutinib** | 0.53% | Intolerance after 1 month | Nilotinib | MR3; intolerance | Imatinib | MR3; intolerance | Asciminib | MR3 |
| 61 | Nilotinib | Primary | 9% | **Imatinib** | 4.7% | No MR1 after 6 months | Bosutinib | No MR2 after 12 months | Ponatinib | No MR3 after 38 months; intolerance | Asciminib | MR3 |

**Legend to Supplementary Table 3a**: resp=response (*BCR::ABL1/ABL1* ratio, RT-qPCR); IS=International Scale; 1L=first line; 2L=second line; 3L=third line; 4L=fourth line; 5L=fifth line; RIP=deceased patient; TRM=transplant-related mortality; CT=chemotherapy; BC=blast crisis; alloSCT=allogeneic hematopoietic stem cell transplant.

**Supplementary Table 3b. Treatment pathways in 4 CML patients with KD mutations at failure of 1L-2GTKI due to resistance.**

| ID | 1L | Resistance | KD Mutation | Resp at switch (IS) | 2L | Best resp to 2L  (IS) | Outcome | 3L  (n=3) | Outcome | 4L  (n=3) | Outcome |
| --- | --- | --- | --- | --- | --- | --- | --- | --- | --- | --- | --- |
| 21 | Dasatinib | Primary | T315I | 10.47% | **alloSCT** | 0 | Molecular relapse post-alloSCT | Ponatinib | Vascular event | Asciminib | MR4.5 |
| 31 | Dasatinib | Secondary (loss of MR4.5, MR4 and MR3) | V299L | 0.61 % | **Nilotinib** | 0 | MR3 loss with new F317L KD mut | Bosutinib | Loss of MR2 and MR1 | Asciminib | MR4.5 |
| 68 | Nilotinib | Secondary  (MR3 loss) | F359I | 0.53 % | **Dasatinib** | 0.5% | Loss of MR2 and MR1 | Ponatinib | Progression into BC | CT | RIP (refractory myeloid BC) |
| 70 | Nilotinib | Primary | G250E | 1.23 % | **Dasatinib** | 0.003% | MR4 |  |  |  |  |

**Legend to Supplementary Table 3b**: resp=response (*BCR::ABL1/ABL1* ratio, RT-qPCR); IS=International Scale; 1L=first line; 2L=second line; 3L=third line; 4L=fourth line; RIP=deceased patient; CT=chemotherapy; BC=blast crisis; alloSCT=allogeneic hematopoietic stem cell transplant; KD=*BCR::ABL1* kinase domain; mut=mutation.

**Supplementary Table 4a. Subsequent TKI changes due to intolerance after 2L-imatinib (n = 10 patients).**

| ID | 1L | Type of  1L-2GTKI intolerance | 2L | Resolved/  Improved  1L-2GTKI AE | 2L AE | 3L | Resolved/Improved 2L-imatinib AE | 3L AE | 4L | Resolved/Improved 3L AE | 4L AE | 5L | Resolved/Improved 4L AE | 5L AE | Current status |
| --- | --- | --- | --- | --- | --- | --- | --- | --- | --- | --- | --- | --- | --- | --- | --- |
| 14 | D | Fatigue | I | Persisting AE and new AE | Fatigue (gr 2), skin rash (gr 2),  abdominal pain (gr 2) | D | Improved (fatigue, gr 1); other AE resolved | none | na | na | na | na | na | na | on TKI |
| 27 | D | Pericarditis | I | Resolved, but new AE | Muscle cramp, arthralgia, nausea and vomiting (all gr 2) | B | Resolved | none | na | na | na | na | na | na | on TKI |
| 38 | D | Rash, itching | I | Resolved, but new AE | Liver enzyme elevation (ALT, gr 4) | N | Improved (Liver enzyme elevation, ALT gr 3) | Liver enzyme elevation (ALT, gr 3) | D | Resolved | none | na | na | na | on TKI |
| 41 | N | Alopecia | I | Resolved, but new AE | Nausea (gr 2) and weight gain (gr 2) | D | Resolved, but new AE | SOB  (gr 3) | B | Resolved, but new AE | Vomiting (gr 2) | I | Resolved | none | TFR |
| 43 | N | Rash | I | Resolved, but new AE | Pneumonitis (gr 3) | D | Resolved, but new AE | Pleural effusion  (gr 2) | B | Resolved | none | na | na | na | on TKI |
| 46 | N | Palpitations, chest pain and HTN | I | Persisting AE and new AE | Neutropenia (gr 3), chest pain (gr 3), arthralgia (gr 2), nausea (gr 2), myalgia (gr 2), mucositis (gr 2) and infection (gr 3) | B | Resolved | none | na | na | na | na | na | na | on TKI |
| 67 | B | Liver enzyme elevation | I | Persisting AE | Liver enzyme elevation (ALT, gr 3) | D | Resolved | none | na | na | na | na | na | na | on TKI |
| 79 | D | SOB | I | Resolved, but new AE | Fatigue, diarrhoea and memory impairment (all gr 2) | B | Resolved | none | na | na | na | na | na | na | on TKI |
| 92 | N | Palpitations and chest pain | I | Resolved, but new AE | Headache (gr 2), myalgia (gr 3) and arthralgia (gr 3) | D | Resolved | none | na | na | na | na | na | na | on TKI |
| 103 | D | Liver enzyme elevation | I | Persisting AE | Liver enzyme elevation (ALT, gr 2) | A | Resolved | none | na | na | na | na | na | na | on TKI |

**Legend to Supplementary Table 4a**: 1L=first line; 2L=second line; 3L=third line; 4L=fourth line; 5L=fifth line; I=imatinib; D=dasatinib; N=nilotinib; B=bosutinib; P=ponatinib; A=asciminib; AE=adverse event; gr=grade (according to CTCAE v.5.0); HTN=hypertension; SOB=shortness of breath; TKI=tyrosine kinase inhibitor; TFR=treatment-free remission; na=not applicable.

**Supplementary Table 4b. Subsequent TKI changes due to intolerance after 2L-2GTKI (n = 6 patients).**

| ID | 1L | Type of  1L-2GTKI intolerance | 2L | Resolved/  Improved  1L-2GTKI AE | 2L AE | 3L | Resolved/  Improved  2L-2GTKI AE | 3L AE | 4L | Resolved/  Improved 3L AE | 4L AE | 5L | Resolved/  Improved 4L AE | 5L AE | 6L | Resolved/  Improved 5L AE | 6L AE | Current status |
| --- | --- | --- | --- | --- | --- | --- | --- | --- | --- | --- | --- | --- | --- | --- | --- | --- | --- | --- |
| 1 | D | GI | B | Resolved (diarrhoea and mucositis both gr 2), but new AE | Liver enzyme elevation (ALT, gr 3) | N | Resolved | none | na | na | na | na | na | na | na | na | na | on TKI |
| 9 | N | PAOD | D | Resolved, but new AE | Insomnia  (gr 2) | B | Resolved | none | na | na | na | na | na | na | na | na | na | TFR |
| 16 | D | Retinal hemorrhage | N | Resolved, but new AE | NIDDM, and conjunctival hemorrhage (gr 3) | I | Resolved, but new AE | Generalised oedema (gr 3), pulmonary oedema (gr 2), malignant hypertension (gr 4) | na | na | na | na | na | na | na | na | na | TFR |
| 58 | N | NIDDM | D | Persistent (NIDDM), and new AE | NIDDM, stomach pain (gr 2) and skin rash (gr 2) | N | Resolved (stomach pain and skin rash), persistent (NIDDM) | NIDDM  (ongoing since 1L-nilotinib) | na | na | na | na | na | na | na | na | na | on TKI |
| 64 | N | Thyroiditis | B | Resolved, but new AE | Liver enzyme elevation (ALT, gr 2) | D | Resolved, but new AE | Memory impairment (gr 2), later improved (gr 1) | na | na | na | na | na | na | na | na | na | on TKI |
| 76 | D | SOB | B | Resolved, but new AE | Diarrhoea (gr 2) | N | Resolved, but new AE | SOB  (gr 2) | I | Resolved, but new AE | Neutropenia (gr 3),  lower GI hemorrhage (gr 2) | P | Resolved, but new AE | Abdominal pain  (gr 2) | A | Resolved | none | TFR |

**Legend to Supplementary Table 4b**: 1L=first line; 2L=second line; 3L=third line; 4L=fourth line; 5L=fifth line; 6L=sixth line; I=imatinib; D=dasatinib; N=nilotinib; B=bosutinib; P=ponatinib; A=asciminib; AE=adverse event; gr=grade (according to CTCAE v.5.0); GI=gastrointestinal; PAOD=peripheral arterial occlusive disease; NIDDM=non-insulin-dependent diabetes mellitus; SOB=shortness of breath; TKI=tyrosine kinase inhibitor; TFR=treatment-free remission; na=not applicable.

**Supplementary Table 4c. Subsequent TKI changes due to intolerance after resistance to 1L-2GTKI (n = 5 patients).**

| ID | 1L | 2L | 2L AE | 3L | Resolved/Improved  2L AE | 3L AE | 4L | Resolved/Improved  3L AE | 4L AE | 5L | Resolved/  Improved  4L AE | 5L AE | Current status |
| --- | --- | --- | --- | --- | --- | --- | --- | --- | --- | --- | --- | --- | --- |
| 61 | N | I | none | B | na | none | P | na | Hypertension (gr 3) | A | Improved | Hypertension  (gr 1) | on TKI |
| 85 | N | D | none | P | na | Neutropenia (gr 3) | AlloSCT | Resolved | na | na | na | na | on TKI  post-alloSCT |
| 87 | D | B | Diarrhoea and fatigue  (both gr 3) | N | Diarrhoea resolved; fatigue persistent (gr 3) | Fatigue  (gr 3) | I | Persistent | Fatigue  (gr 3) | A | Resolved | none | on TKI |
| 96 | D | P | Liver enzyme elevation (ALT, gr 3) | D | Resolved | none | A | na | none | na | na | na | on TKI |
| 106 | D | P | Thrombocytopenia (gr 4) | A | Resolved | none | na | na | na | na | na | na | on TKI |

**Legend to Supplementary Table 4c**: 1L=first line; 2L=second line; 3L=third line; 4L=fourth line; 5L=fifth line; I=imatinib; D=dasatinib; N=nilotinib; B=bosutinib; P=ponatinib; A=asciminib; AE=adverse event; gr=grade (according to CTCAE v.5.0); TKI=tyrosine kinase inhibitor; alloSCT=allogeneic hematopoietic stem cell transplant; na=not applicable.

**Supplementary Table 5. Adverse event profile in patients receiving 3/4GTKI.**

Out of 45 CML patients who failed their 1L-2GTKI, a total of 12 (26.6%) and 10 (22.2%) patients received ponatinib and asciminib pre-alloSCT, respectively. Of the latter, five patients (50%) had previously received ponatinib. Two patients received 3/4GTKI post-alloSCT: one had asciminib and the other had ponatinib followed by asciminib.

In the pre-alloSCT setting, five of 12 patients (41.6%) suffered from adverse events which led to discontinuation of ponatinib. None of those who received asciminib had to interrupt the drug due to intolerance. Supplementary Table 5 provides the detailed description of the adverse events in this subgroup of patients.

| ID | Patient group | TKI  (P or A) | Line of therapy | Current TKI dose or last TKI dose before interruption | AE grade ≥ 2  or  CVE | TKI discontinuation on account of AE |
| --- | --- | --- | --- | --- | --- | --- |
| 48 | Intolerant (2L-imatinib) | P | 4 | 30mg OD | none | na |
| 48 | Intolerant (2L-imatinib) | A | 5 | 40mg BD | none | na |
| 82 | Intolerant (2L-imatinib) | A | 4 | 40mg OD | none | na |
| 103 | Intolerant (2L-imatinib) | A | 3 | 40mg OD  alt. days | none | na |
| 76 | Intolerant (2L-2GTKI) | P | 5 | 15mg OD  alt. days | Nausea and vomiting  (both gr 2) | **YES** |
| 76 | Intolerant (2L-2GTKI) | A | 6 | 40mg OD alt. days > TFR | none | na |
| 20 | Res | P | 2 | 15mg OD | none | na |
| 93 | Res | P | 2 | 45/30mg OD  alt. days | Hypertension (gr 2) | NO |
| 96 | Res | P | 2 | 30mg OD | Liver enzyme elevation  (ALT, gr 3) | **YES** |
| 96 | Res | A | 4 | 80mg OD | none | na |
| 106 | Res | P | 2 | 15mg OD  alt. days | Thrombocytopenia (gr 4) | **YES** |
| 106 | Res | A | 3 | 80mg OD | none | na |
| 22 | Res | P | 3 | 30mg OD | none | na |
| 65 | Res | P | 3 | 30mg OD | none | na |
| 66 | Res | P | 3 | 15/30mg OD alt. days | Weight gain (gr 3) | NO |
| 74 | Res | A | 3 | 80mg OD | none | na |
| 85 | Res | P | 3 | 15mg OD | Neutropenia (gr 3) | **YES** |
| 85 | Res | A  post-  alloSCT | 5 | 40mg OD  alt. days | none | na |
| 87 | Res | A | 5 | 40mg OD | none | na |
| 61 | Res | P | 4 | 15/30mg OD alt. days | Hypertension (gr 3) | **YES** |
| 61 | Res | A | 5 | 40mg BD | Hypertension (gr 1) | NO |
| 31 | Res (*BCR::ABL1* KD mut) | A | 4 | 80mg OD | Palpitations (gr 2) | NO |
| 21 | Res (*BCR::ABL1* KD mut) | P  post-alloSCT | 3 | 15/7.5mg OD alt. days | Renal artery stenosis (gr 2) | YES |
| 21 | Res (*BCR::ABL1* KD mut) | A  post-  alloSCT | 4 | 20mg OD | Thrombocytopenia (gr 3) | NO |
| 68 | Res (*BCR::ABL1* KD mut) | P | 3 | 15mg OD  alt. days | none | na |

**Legend to Supplementary Table 5**: P=ponatinib; A=asciminib; AE=adverse event; gr=grade (according to CTCAE v.5.0); Intolerant (2L-imatinib)=patients who failed 1L-2GTKI due to intolerance and were switched to 2L-imatinib; Intolerant (2L-2GTKI)=patients who failed 1L-2GTKI due to intolerance and were switched to 2L-2GTKI; Res= patients who failed 1L-2GTKI due to resistance and a *BCR::ABL1* KD (kinase domain) mutation was not detected; Res (*BCR::ABL1* KD mut)=patients who failed 1L-2GTKI due to resistance and a *BCR::ABL1* KD (kinase domain) mutation was detected; alloSCT=allogeneic hematopoietic stem cell transplant; OD=once a day; BD=twice a day; alt. days=on alternate days; TFR=treatment-free remission; TKI=tyrosine kinase inhibitor; CVE=cardiovascular event; na=not applicable.
